# pH-Dependent Conformational Changes in Proteins and Their Effect on Experimental pK_a_s: The Case of Nitrophorin 4

**DOI:** 10.1371/journal.pcbi.1002761

**Published:** 2012-11-01

**Authors:** Natali V. Di Russo, Dario A. Estrin, Marcelo A. Martí, Adrian E. Roitberg

**Affiliations:** 1 Quantum Theory Project and Department of Chemistry, University of Florida, Gainesville, Florida, United States of America; 2 Departamento de Química Inorgánica, Analítica y Química Física/INQUIMAE-CONICET, Facultad de Ciencias Exactas y Naturales, Universidad de Buenos Aires, Ciudad Universitaria, Buenos Aires, Argentina; 3 Departamento de Química Biológica, Facultad de Ciencias Exactas y Naturales, Universidad de Buenos Aires, Ciudad Universitaria, Buenos Aires, Argentina; University of Houston, United States of America

## Abstract

The acid-base behavior of amino acids is an important subject of study due to their prominent role in enzyme catalysis, substrate binding and protein structure. Due to interactions with the protein environment, their pK_a_s can be shifted from their solution values and, if a protein has two stable conformations, it is possible for a residue to have different “microscopic”, conformation-dependent pK_a_ values. In those cases, interpretation of experimental measurements of the pK_a_ is complicated by the coupling between pH, protonation state and protein conformation. We explored these issues using Nitrophorin 4 (NP4), a protein that releases NO in a pH sensitive manner. At pH 5.5 NP4 is in a closed conformation where NO is tightly bound, while at pH 7.5 Asp30 becomes deprotonated, causing the conformation to change to an open state from which NO can easily escape. Using constant pH molecular dynamics we found two distinct microscopic Asp30 pK_a_s: 8.5 in the closed structure and 4.3 in the open structure. Using a four-state model, we then related the obtained microscopic values to the experimentally observed “apparent” pK_a_, obtaining a value of 6.5, in excellent agreement with experimental data. This value must be interpreted as the pH at which the closed to open population transition takes place. More generally, our results show that it is possible to relate microscopic structure dependent pKa values to experimentally observed ensemble dependent apparent pK_a_s and that the insight gained in the relatively simple case of NP4 can be useful in several more complex cases involving a pH dependent transition, of great biochemical interest.

## Author Summary

The interaction of an amino acid with its protein environment can result in an acid-base behavior that is very different from what would be observed in solution. This environment can be greatly altered when the protein changes conformation. As a result, the amino acid will have two different “microscopic” pK_a_ values. Nitrophorin 4 is a good case study to explore this behavior, because it undergoes a pH-dependent conformational change that is well characterized experimentally. Using computer simulation tools, we found that the key titratable Aspartic acid 30, has two very different microscopic pK_a_s: 4.3 and 8.5, which are significantly different to the observed transition pK_a_ in solution. However, using a simple model, we were able to understand how this causes the conformational change to take place at pH∼6.5, as measured experimentally. The insight gained in this relatively simple case can be useful in other more complex cases where the apparent pK_a_ is also a result of the interplay of different conformations where some amino acids experience very different environments.

## Introduction

Ionizable amino acid residues have been shown to play important roles in the binding of proteins to other molecules and in enzyme mechanisms. They also have a large influence on protein structure, stability and solubility [1], [2]. The types of interactions these side chains will have with their environment depend on their protonation state. Because of this, their pK_a_ values and the factors that influence them are a subject of intense biochemical interest. Strongly altered pK_a_ values are often seen in the active sites of enzymes, to enhance the ability of ionizable residues of acting as nucleophiles, electrophiles or general bases and acids [3]. As a consequence of the change in protonation of these residues, the stability of proteins is pH-dependent [4]. Changes in intracellular pH have been shown to regulate essential processes like cell proliferation and apoptosis. However, our understanding of how changes in environmental pH affect proteins is limited [5].

The pK_a_ values of ionizable residues in folded proteins can be strongly influenced by the local environment. The three main factors affecting these values are charge-charge interactions [6], charge-dipole interactions, which include hydrogen bonding and the interaction with macroscopic helix dipoles [3], [7], and the Born effect (dehydration) [8], [9]. The effect of charge-charge and charge-dipole interactions on the pK_a_ value becomes stronger when the dielectric constant decreases, as in the case of the hydrophobic interior of proteins [10].

Through these effects, the environment can alter the pK_a_ of a residue to varying extents. For example, while the average pK_a_ value measured in proteins for Asp is 3.5±1.2, values ranging from 0.5 to 9.2 have been reported for this residue [1]. Usually, these values are measured using nuclear magnetic resonance (NMR) by fitting chemical shift vs. pH curves. In some cases they are obtained indirectly by measuring other properties, such as equilibrium constants [11] or midpoint potentials [12] as a function of pH. However, changing the pH can modify the protonation state of a residue, which can couple with a conformational change that causes the environment of this residue to change [2], [13], possibly altering its pK_a_ value. Tanford has shown that, if the equilibrium between two conformations of a protein is pH dependent, then at least one titratable group must have a different pK_a_ in the two conformations [14]. In this manuscript, we will refer to these as “microscopic” pK_a_s. Experimentally, usually only one apparent macroscopic pK_a_ is measured for each residue, which cannot be directly assigned to any individual microscopic conformation.

The coupling of protonation and conformational equilibria is one particular example of the concept of thermodynamic linkage, which was first introduced by Wyman to describe the Bohr effect [15]. Other work in this area includes the study of protein denaturation by pH [16], the development of methods that can accurately treat the coupling between multiple titration sites [17], [18], and the improvement of pK_a_ calculations by taking into account the coupling between conformational flexibility and ionization states [19], [20]. In particular, García-Moreno has studied the linkage between local conformational changes and proton binding in SNase both experimentally [21] and computationally, using an ensemble-based description [22].

Nitrophorin 4 (NP4) is a system displaying this complexity and we will use it as a case study to explore the relationship between the microscopic and macroscopic “apparent” pK_a_s. We will show how two distinct microscopic pK_a_s can give rise to a macroscopic pK_a_ in the physiologically relevant range of 6.5–7.5. Scenarios like NP4 occur in several systems of high biochemical interest where the insight obtained for the relatively simple case of NP4 can be applied. Some of these will be discussed in more detail in the discussion section of this manuscript.

Nitrophorins (NPs) are nitric oxide (NO) carrier heme proteins found in the saliva of some blood-sucking insects [23]. The most extensively studied NPs are those found in the salivary glands of the kissing bug, *Rhodnius prolixus*, which can act as a vector of the parasite *Trypanosoma cruzi*, the causative agent of Chagas' Disease (American Trypanosomiasis) [24]. Up to seven of these NPs have been cloned [25] and spectroscopically, kinetically [11] and structurally [26]–[29] characterized. Among these, NP4 is the most extensively studied. In order to fulfill its biological role, NP4 must retain NO in the insect's saliva and release it in the victim's tissues. This selectivity is achieved by binding NO in a pH sensitive manner. The protein binds NO tightly inside the salivary glands of the bug, where the pH is approximately 5. However, when it is injected into the victim's tissues, which have a pH of approximately 7.4, NP4 undergoes a conformational change that reduces substantially its affinity for NO [11], [30].

**Figure 1:**
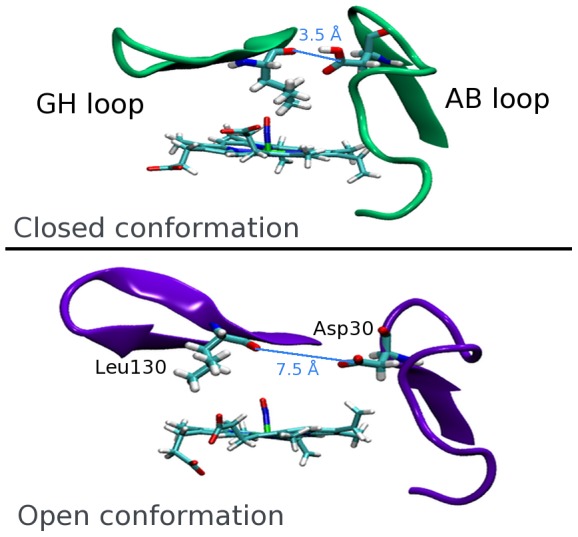
Structure of the AB and GH loops in the open and closed conformations. The position of Asp30 and Leu130, and the distance between these residues is also shown. The open conformation is predominant at pH 7.5, while the closed structure is predominant at pH 5.5.

NP4 is a good case for studying pH-dependent conformational changes using computational tools because there are NO-bound and unbound crystallographic structures available of both the low pH and the high pH state [31] (referred to as “closed” and “open” structures, respectively) which show that this conformational change involves mainly loops AB and GH, which cap the heme side of the β barrel to which NO is bound [31], [32] (Figure 1 and Figure S1). Furthermore, the pH-dependent conformational change that leads to NO release has been studied both theoretically and experimentally [31]–[34]. In this context, although it has been shown that the strength of the heme-NO bond is pH-independent [11], results from our group show that NO is only able to escape in the open conformation because in the low pH state the kinetic escape barrier is very high [33].

Previous studies have also shown that the protonation of Asp30, which is conserved in the four major NPs of adult insects [12] plays an important role in the conformational change. In the closed conformation this residue is protonated and interacting with Leu130 through a hydrogen bond, keeping loops AB and GH close together. This interaction is lost in the open structure, in which Asp30 is ionized and becomes solvent exposed [32], [35] (Figure 1). In earlier work we have shown that setting the protonation state of this key residue is enough to maintain a closed or open conformation during extensive molecular dynamics (MD) simulations [35]. Although much has been learned about the way NPs work, the fundamental question of what is the value for the Asp30 pK_a_, and how it is related to the conformational change remains unanswered.

The first attempt to measure a pK_a_ value in NP4 was derived from NO affinity studies as a function of pH. The observed NO dissociation equilibrium constant (K_d_NO) shows a simple sigmoidal behavior when the pH is changed from 5.0 to 8.0, allowing the determination of an observed apparent pK_a_ from the NO binding data of 6.5 [11]. Later, studies tried to focus on particular residues using a combined site directed mutagenesis (changing the titratable residues for alanines) and electrochemical approach. The resulting apparent pK_a_ value of Asp30 in NP4 was estimated to be 5.5±0.4 [12].

In this work we have used constant pH MD (CpHMD) [36] to study the titration of Asp30 in NP4 in order to determine its microscopic pK_a_s and relate them to the NP4 conformational transition that governs NO affinity. Traditionally, the treatment of pH in MD has been limited to setting a fixed protonation state for each titratable residue, but there are several problems related to this approach: (a) the pK_a_ of a given residue may be unknown, (b) if the pK_a_ of one residue is similar to the solvent pH, a single protonation state is insufficient to appropriately describe the ensemble of protonation states that will be observed at that pH, (c) it is not possible to study the coupling between pK_a_, protonation state and conformation. A significant advantage of the CpHMD method is that by using MD, the method allows the sampling of different conformations. This is very important because pK_a_ calculations tend to be highly sensitive to details of the environment around the residue that is being titrated. Methods that incorporate little to no conformational flexibility suffer from problems like, for example, exaggerating the magnitude of electrostatic effects, which results in extreme pK_a_s [4], [19]–. CpHMD makes it possible to study the coupling between the solvent pH, the microscopic pK_a_s and the conformation. Given that the pH-dependent conformational change of NP4 is relatively large, an implicit solvation model will be used to ensure that the relevant conformational space will be sampled during the MD simulation. Other possible strategies to deal with this issue include driving the conformational change using Umbrella sampling or an overcharging strategy [38], [39].

The CpHMD [36] method allows titratable residues to be in any discrete protonation state during the computational simulation. Every 10 MD steps, a Monte Carlo (MC) sampling scheme is used to determine the protonation state based on the energy differences between the possible protonation states [36], [40]–[41]. The pKa of the amino acid residue can be estimated based on the fraction of time it spent protonated at a given pH, assuming ergodicity and Henderson-Hasselbach titration curves [36]. A slightly different approach for performing constant pH MD simulations allows protonation parameters to vary continuously between protonation states [42]–[44].

Our results show that the microscopic pK_a_ of Asp30 in the closed conformation (pK_a_ = 8.5) is significantly different from that in the open conformation (pK_a_ = 4.3). Furthermore, we also show that the protein conformation and, therefore, the pK_a_ of Asp30 are closely coupled to the solvent's pH, and we trace how the pK_a_ changes as the conformational change takes place. With this data, we performed an analysis similar in spirit to Tanford's [14] but with a focus on how the microscopic pK_a_ values of this key residue determine the macroscopic, observed pK_a_. We were able to set limits for the free energy values for the conformational closed-to-open transition for both protonation states of Asp30 and calculate the pH at which the closed to open transition takes place. The computed value, which is close to 6.5, is in excellent agreement with the apparent pK_a_ obtained for the experimental titration of NO affinity. We determined that the apparent pK_a_ corresponds to the pH at which the conformational change takes place. The analysis performed and the conclusions drawn for the case of NP4 can be applied to several more complex cases of great biochemical interest.

## Results

The results are organized as follows. First, we will describe simulations of the open and closed structure at the solvent pH values at which they are stable. From these we were able to estimate the microscopic pK_a_ of Asp30 in both conformations. We will then describe the results from simulations in which each structure was placed at a solvent pH at which it is not stable, focusing on how the conformational change is coupled to the microscopic pK_a_ of Asp30. Finally, in the discussion we will show how the microscopic pK_a_s obtained for each conformation are related to the macroscopic pK_a_.

### Stable simulations: Closed conformation at pH 5.5 and open conformation at pH 7.5

As expected, CpHMD simulations of the closed conformation at pH = 5.5 and of the open conformation at pH = 7.5 show that both structures remain stable during the simulation timescale. In our previous studies we were able to determine some key interactions that characterize each protein conformational state (open or closed) and that remain stable during fixed Asp30 protonation state simulations [35]. Distance CAsp30-OLeu130 shows whether the hydrogen bond that keeps the AB and GH loops close together is formed. Distances CBVal36-CGLeu130 and CGAsp35-OAsp129 can be monitored to know whether the NO escape route is open (Figure S2). As can be seen from Table 1, the results for the CpHMD simulations show that the interactions are maintained to similar values as those obtained previously in ref. 35.

**Table 1:** Average distances for selected characteristic interactions in the open and closed conformations of NP4.

Structure	dCAsp30-OLeu130	dCBVal36-CGLeu130	dCGAsp35-OAsp129
**Closed, fixed** (*)	3.6±0.3	5.9±0.7	5±1
**Closed, pH 5.5**	3.5±0.3	6.4±0.8	5±1
**Open, fixed** (*)	7.2±0.9	13±1	12±2
**Open, pH 7.5**	8±1	14±2	14±3

Distances and their respective standard deviations are shown in Å.

(*) Data from structures labeled “fixed” corresponds to simulations where the protonation state of Asp30 is fixed, while in the rest of the cases the protonation of Asp30 is allowed to change.

A detailed analysis of Asp30 shows that in the simulations of the closed state at a solvent pH of 5.5, Asp30 remains protonated during most of the simulation, resulting in a pK_a_ value of 8.5. The simulations of the open state at solvent pH = 7.5 show that Asp30 remains ionized most of the time, resulting in a pK_a_ value of 4.3. Therefore, the pKa of Asp30 in the closed conformation is significantly different from that in the open conformation. The obtained pKa values can be rationalized looking at the Asp30 interactions along the MD. In the open state the residue is solvent exposed, with a solvent-accessible surface area (SASA) value of 40±2 Å^2^ and, therefore, has a pKa value close to 4.0, the pK_a_ for an isolated Asp [3]. In the closed state, the residue is almost completely buried (the SASA value is reduced to 9±4 Å^2^) and involved in a hydrogen bond. As a consequence, the resulting pKa is 4.5 units higher than the intrinsic Asp pK_a_. Although this is a large pK_a_ shift, even higher pK_a_s have been reported for Asp in the literature [1].

### Transition inducing simulations: Closed conformation at pH 7.5 and open conformation at pH 5.5

**Figure 2:**
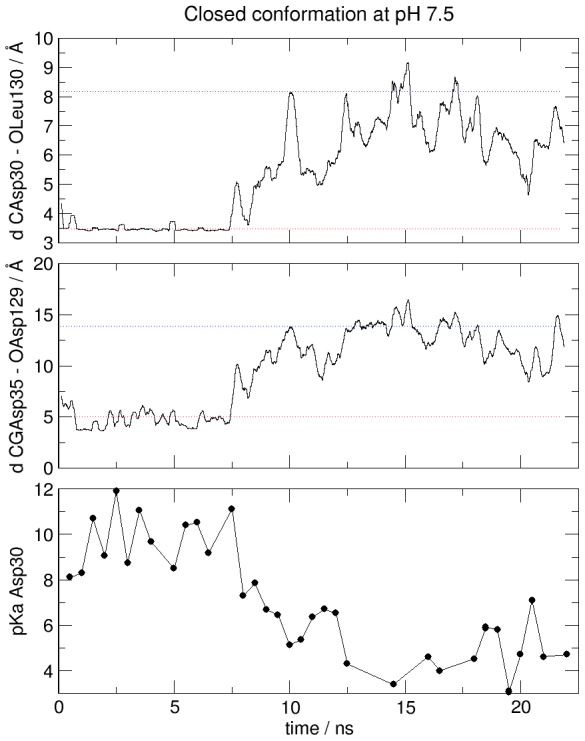
Time evolution of relevant parameters when closed NP4 is placed at a solvent pH of 7.5. Top: Running average of distance Asp30-Leu130; Middle: Running average of distance Asp35-Asp129; Bottom: Asp30 microscopic pK_a_. The average values of these distances in the stable simulations of the closed (red) and the open (blue) structures are also shown.

We now turn our attention to CpHMD simulations that start from the closed conformation and are run at pH = 7.5 and that of the open conformation at pH = 5.5, i.e. corresponding to conditions where the starting structure is not optimal. As expected, results show that these structures are not stable under these conditions (see RMSD values in Figure S3). In both cases the CpHMD scheme changes the protonation state of Asp30 during the dynamics and this triggers the onset of the conformational change, as evidenced by the shift in the monitored distances towards the typical values of the opposite structure, i.e. the stable structure at the simulation pH. It is important, then, to highlight that using this method we were not only able to measure the pK_a_s, but also to observe the conformational change that takes place upon a change in protonation of Asp30. As consequence of the onset of the structural transition, the microscopic pKa changes along the simulation, a fact that is also accompanied by a change in the SASA of the residue (Figure S4). Figure 2 illustrates this point, by showing the time evolution of relevant distances and of the pK_a_ as a function of time when the closed conformation is placed in pH 7.5 solvent. A close look at the Asp30 interactions shows that ionization of this residue causes the hydrogen bond between Asp30 and Leu130 to break after 9 ns of simulation time. This change is coupled to the opening of the NO escape route, as shown by the increase in the distance Asp35-Asp129, which reaches values that are close to those expected in the open conformation. The RMSD plots of the AB and GH loops with respect to the closed and open structure (Figure S3), also show that the position of these loops changes to resemble that of the open conformation.

On the other hand, for the simulations which start from the open conformation but are placed at pH 5.5, as Asp30 becomes protonated, its side chain is rapidly buried inside the protein (SASA is reduced), bringing it close to Leu130 and allowing the hydrogen bond between these residues to form, closing the NO escape route. Also in this case it is possible to see that as the conformational change occurs, Asp30 pK_a_ increases to reach values typical of the closed conformation (for additional detail, see Figure S5 and text S1).

In summary, our results show that the microscopic pK_a_ of Asp30 in the open conformation (4.3) is significantly different from that in the closed conformation (8.5). These pK_a_ values enable the protein to be stable in its closed conformation at a low pH, and the open conformation to be stable at a high pH, as required to fulfill its biological role. Transition-inducing CpHMD simulations enabled us to show that when a conformation is placed at a pH at which it is not stable, a conformational change takes place and, as this happens, the pK_a_ of Asp30 changes in time.

## Discussion

### The relationship between the microscopic and apparent macroscopic pK_a_ values

**Figure 3:**
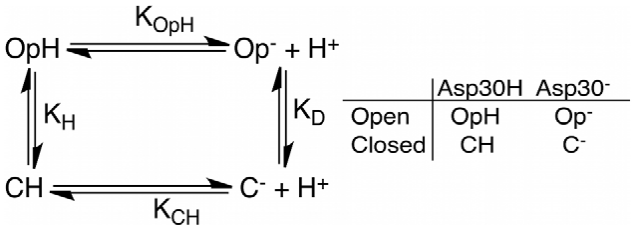
Thermodynamic cycle relating the relevant chemical species and their equilibrium constants.

We have shown that there are two distinct microscopic pK_a_s, 4.3 and 8.5, with neither corresponding to the experimentally observed estimated value assigned to Asp30 of 5.5±0.4, as derived from electrochemical measurements [12], or the apparent pK_a_ of 6.5 derived from the pH dependence of the NO binding equilibrium constant [11]. The obvious question now is: Where does the observed pK_a_ come from? To relate the computed microscopic pK_a_ values to the experimental observations, and to understand how the NP4 population ensemble changes with the solvent pH, we postulate a simple four state system (similar to Tanford's [14]). We analyzed the relative populations of the different NP4 states according to the chemical equilibria shown in Figure 3.

**Figure 4:**
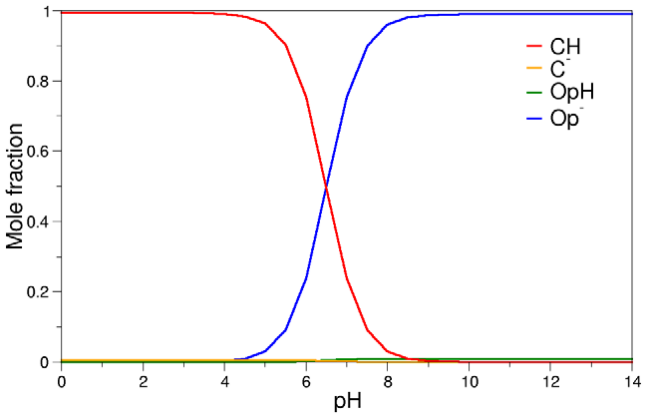
Mole fractions of the relevant chemical species as a function of pH. K_D_ = 100, K_H_ = 6.31×10^−3^.

According to Figure 3, the equilibrium between the open state with Asp30 protonated (OpH) and deprotonated (Op^−^) is described by the computed pK_a_ in the open conformation (pK_OpH_) value of 4.3. The corresponding value in the case of the closed conformation is pK_CH_ = 8.5. The values of K_H_ = (OpH)/(CH) and K_D_ = (Op^−^)/(C^−^) correspond to the equilibrium constants determined by the free energy difference between the open and closed conformations and with Asp30 neutral or charged, respectively. Although these values are unknown, reasonable bounds can be estimated. In order to function efficiently, at low pH NP4 must be predominantly in the closed state, but since Asp30 is mostly protonated, for this to be true, K_H_≪1. On the other hand, at a high pH, the open structure must be predominant. Since at this pH Asp30 is found deprotonated, it follows that K_D_≫1. However, as shown by Figure 3, both K_H_ and K_D_ are not independent. Using the thermodynamic cycle, both equilibrium constants can be related by equation 1:
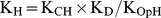
(1)Using Equation 1, K_H_ for example can be calculated if all other constants are known, leaving K_D_ as the only unknown parameter. Using the cycle above, mass balance equations and the values of pK_CH_ and pK_OpH_ obtained from our calculations, the equilibrium concentrations of all the relevant chemical species can be calculated at any pH value for any given value of K_D_ (see text S2 for complete derivation). Figure 4 is an example of this, in which K_D_ was chosen to be 100 and, as a consequence, K_H_ equals 6.31×10^−3^.

**Figure 5:**
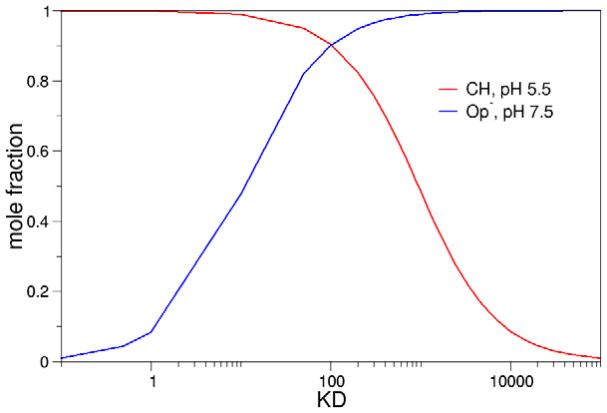
Concentration of CH at pH 5.5 and Op^−^ at pH 7.5 as a function of KD. (CH) at pH 5.5 shown in red, while (Op^−^) at pH 7.5 is shown in blue. The concentrations are given as mole fractions.

Figure 4 shows that at a pH of less than 5, the closed conformation in which Asp30 is protonated is predominant. Raising the pH results in a dramatic increase of the concentration of the open conformation with Asp30 ionized, which is the predominant chemical species at pH higher than 8. These results are consistent with the kinetic and crystallographic evidence available. It is important to note that using the pK_OpH_ and pK_CH_ values obtained in the present work, the diagram will show the same behavior only if 10<K_D_<1500. For values outside this range, either the concentration of OpH or of C^−^ become significant (>10%) at extreme pH values, contradicting the known experimental data. It is also possible to calculate what is the K_D_ value that maximizes the efficiency of the protein to fulfill its biological role. Figure 5 shows how the concentration of CH at pH 5.5 and Op^−^ at pH 7.5 varies as a function of K_D_. NP4 will be most efficient by simultaneously maximizing the concentration of the closed form at pH 5.5 and the concentration of the open form at a high pH, to ensure that NO is released in the victim's tissues and not in the salivary glands of the insect. This is achieved when K_D_ is approximately 100 Larger values of K_D_ result in an increase of the population of OpH at a low pH, which would cause release of NO in the insect's salivary glands while lower values would cause an increase in the population of C^−^ at high pH, which would reduce the amount of NO released into the victim's tissues. It is important to note that using this K_D_ value also yields results which are in agreement with the observation that at pH 5.5 and 7.5 there is a mixture of the open and closed conformations [45], [46]: the population of the open conformation at pH 5.5 and of the closed conformation at pH 7.5 are approximately 10%. Studies of CO rebinding upon laser photolysis show that the population of the closed conformer at neutral pH is significant [47], and estimated to be 23% [48]. This is also in good agreement with our results: for K_D_ = 100 the closed population is 24%.

**Figure 6:**
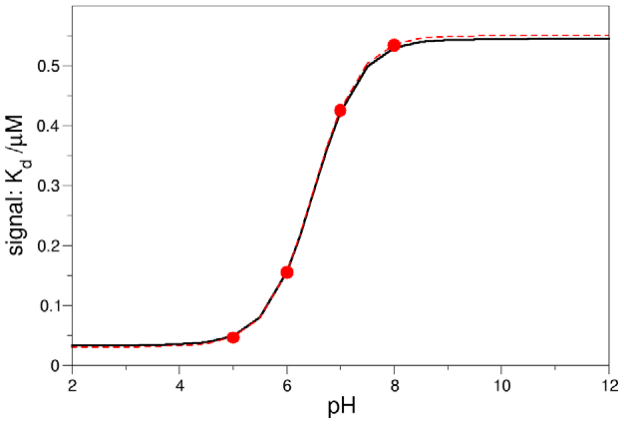
Calculated signal as a function of pH, and comparison to experimental results. Black: Signal, as defined in Equation 2, as a function of pH for K_D_ = 100, K_H_ = 6.31×10^−3^, α = 0.03, β = 0.55 (α and β are obtained from ref. 11). Red: NO dissociation equilibrium constant (K_d_NO) vs pH and fit to the equation of a titration curve with an apparent pK_a_ of 6.5, as obtained from ref. 11.

**Figure 7:**
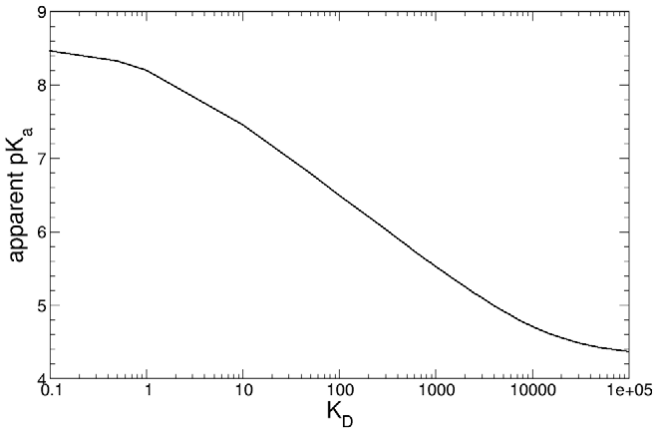
Apparent pK_a_, calculated using Equation 3, as a function of K_D_.

As the experiments designed to measure the pK_a_ typically report on the overall protein conformation, we can calculate the observed signal (i.e. spectroscopic signature, redox potential or any other conformation-dependent measurable property) according to Equation 2.
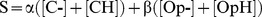
(2)In this equation, α and β are parameters that correspond to the limiting values of the measured signal, in the closed and open conformations respectively. Equation 2 can be applied, for example, in the case where the measured property is the NO dissociation equilibrium constant, K_d_NO, in NP4. Using the computed pK_a_ values, K_D_ = 100 and obtaining α and β from the limits of the K_d_NO vs. pH plot taken from ref. 11 we can plot the signal as a function of pH, as shown in Figure 6. This plot has the expected sigmoidal shape and is in excellent agreement with the experimental data. The apparent pK_a_ can be calculated as the inflection point of this curve according to Equation 3 (see Text S2 for more detail), yielding a value of 6.5, also in excellent agreement with the reported data [11].
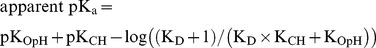
(3)From Equation 3, it follows that the pK_a_ is always bounded by the values of the pK_a_ in the open and closed conformations, as shown in Figure 7. Moreover, varying K_D_ in the range of values discussed above does not significantly change the population transition pKa, but only the [OpH]/[CH] and [Op^−^]/[C^−^] ratios at high or low pH values, underscoring the reliability of the obtained results and at the same time providing bounds for the transition free energies.

Finally, we analyzed the dependence of the apparent pK_a_ on the estimated K_D_ value, as shown in Figure 7. The results show that for a K_D_ in the range 10<K_D_<1500 the apparent pK_a_ varies only between 5.4 and 7.5, showing that our results allow us to predict the experimental apparent pK_a_ within one pK_a_ unit, having only made broad assumptions regarding the value of K_D_. However, using the arguments presented in Figure 5, we can see that a K_D_ of approximately 100 gives an apparent pK_a_ value in very good agreement with the experimental results based on NO equilibrium [11]. A higher K_D_ (K_D_≈1000), is necessary to obtain a lower apparent pK_a_ of 5.5, consistent with the estimate for Asp30 [12].

At this point it is very important to note that although the microscopic structure dependent pK_a_ values are assigned to a particular residue, Asp30, the apparent pK_a_ which is related to the experiment is not necessarily the apparent pK_a_ value for this residue, but the pK_a_ of the structural transition, which as will be discussed below is what is usually measured. Moreover, although in the present work only Asp30 was set as titratable, the choice is justified by the facts that a) differentially setting this residue protonation state is enough to maintain each conformation during the MD simulations [35] b) the resulting apparent pK_a_ derived from the computed values is in excellent agreement with the experimental available data. Moreover, the fact that changing the protonation state of Asp30 is enough to describe with reasonable accuracy the system behavior, although it does not rule out the possibility that other titratable residues contribute to the observed process, strongly suggests, in agreement with experimental data, that it plays a primary role.

The type of analysis presented here to relate the microscopic pK_a_ values to the apparent pK_a_ can be applied to other proteins that undergo pH-dependent conformational changes and may provide useful insight for understanding more complex cases, where several titratable residues interact.

### Relationship to other systems

Our results may explain why titrations of several aspartic and glutamic acid residues in NP1, NP2 and NP4 (including Asp30) yield values in the 5.5–7.5 range [12]. Specifically, NP2 undergoes a pH-dependent conformational change that is very similar to NP4, with Asp29 playing a key role similar to that of Asp30 [49]. The experimentally measured pK_a_ for NP2Asp29 is 5.4±0.4 [12]. The arguments presented here can be extended to that case and a small change in each microscopic pK_a_ or the K_D_ would result in the observed macroscopic value. It is likely that they can also be extended to the recently crystallized NP7, in which the key aspartic acid residue is also conserved [50]. Also, β-lactoglobulin displays a pH-dependence similar to that observed in NPs. The protein undergoes a conformational change, known as the Tanford transition, at pH∼7.5 [51] that is coupled to a change in the protonation state of Glu89 [52]–[53]. Tanford used a setup similar to ours to show that the microscopic pK_a_ of this residue must be different in the two conformations [14].

Several proton translocation pathways in active transporters have functionally important carboxylates with pK_a_ values close to physiological pH for at least some stage of the transport cycle [54]. For example, in the multidrug transporter AcrB, protonation and deprotonation events trigger conformational changes that allow drug transport. Two essential Asp residues (Asp407 and 408) are responsible for proton translocation. In one of the conformations, these are considered to be deprotonated and interacting with a positively charged Lys side-chain. However, when the carboxylates become protonated, Lys turns away from them and towards a Thr residue [55]. In the context of our analysis, the experimentally measured pK_a_ value of 7.4 for Asp408 can be interpreted as arising from two very different microscopic pK_a_s coupled by a conformational change. Another case where carboxylates play a key role is the Nha Na^+^/H^+^ antiporter of *E. coli*. In the pH = 4 structure multiconformation continuum electrostatics calculations predict that Asp 163 and 164, which are part of the Na^+^ binding site, have a pK_a_ of >15. However, a structural change that lowers the pK_a_ of these residues is required for the antiporter to be active at its normal pH range of 6.5–8.5 [56]. Finally, the type of analysis presented in this work could be useful to interpret the pK_a_ value of ∼7 measured for Glu65 [57]–[59], the residue thought to interact with the key Arg (known as stator charge) at the a/c interface of F_1_F_0_ ATP synthase. The current model for torque generation in this system involves Arg forming an ion pair with a deprotonated Glu65. Upon competition with the coupling ion, Arg is displaced and Glu65 becomes protonated, triggering a conformational change that buries this residue in a hydrophobic region. Based on the environment of Glu65 in each state, we expect there will be two very different microscopic pK_a_s. However, the measured pK_a_ of 7 is interpreted in some of the work on this topic as arising from a hydrophobic environment and a local structure around this residue that has been optimized to raise the pK_a_ to the physiological range [58]–[59]. We want to emphasize that in cases such as this it is crucial to think of the measured pK_a_ as arising from the coupling of two structures and not from a single static structure.

### Can microscopic pK_a_s be observed experimentally?

Our results also suggest the possibility that the microscopic pK_a_s cannot directly be measured experimentally, but can certainly be computed via a thermodynamics model like ours. Typically, the experiments involve the acquisition of data (e.g. NMR, IR, enzymatic rates, equilibrium binding constants) as a function of solvent pH over the range of interest and subsequent fitting of the data to a titration curve [60]. These are structural probes and, as such, they do not report directly on protonation states. In our system, the following paradox applies: to be able to accurately measure the pK_a_ of Asp30 in the closed structure it would be necessary to make measurements at a solvent pH close to the residue's pK_a_ of 8.5. However, at this pH the protein is found in its open conformation, so the experiment seems impossible to perform. A similar reasoning applies to the measurement of the pK_a_ in the open conformation. Only in some particular cases it is possible to obtain the value of the microscopic pK_a_s of one residue in two different conformations using pH-jump experiments [61]–[64]. Using a thermodynamic cycle similar to the one shown in Figure 3 it is possible to obtain a relationship between the relaxation time of the experiment and the pK_a_s. Knowledge of microscopic pK_a_s can be used to improve the analysis of NMR spectra tracking a pH-induced conformational change [2]. It is also important to keep in mind that pH-dependent conformational changes may be taking place when comparing data from pK_a_ calculations to experimental results [44].

### Conclusion

Our results show that there are two distinct microscopic Asp30 pK_a_ values: 8.5 in the closed conformation and 4.3 in the open conformation. These values are reasonable once the environment of Asp30 in each state is considered: in the closed structure it is buried and forming a hydrogen bond with Leu130 that is key to keeping the AB and GH loops close together, while in the open structure this bond is broken and the residue becomes exposed to the solvent.

We were also able to confirm that the closed structure is stable at a pH of 5.5, while the open structure is stable at pH 7.5. However, we found that the closed structure, when studied at a high pH, spontaneously undergoes a conformational change to the open structure. Similar results were obtained when the open structure was placed at a low pH. We found that these transitions are coupled to a change in the protonation state of Asp30 and that they can be traced by analyzing the change in the distances between amino acids significantly involved in the process and in the microscopic pK_a_ of Asp30. These results show that the microscopic pK_a_ of Asp30 is highly coupled to the conformation of NP4, which in turn is pH-dependent.

Finally, we were able to reconcile the two microscopic pK_a_s obtained with the apparent pK_a_ measured experimentally. Our results show that the apparent pKa that governs the structural transition is 6.5, in excellent agreement with the pK_a_ obtained from NO affinity studies. The apparent pK_a_ corresponds to the pH at which the conformational change from the closed to the open state takes place. It is important to highlight that while the microscopic pK_a_s were significantly different from the apparent pK_a_, the coupling between the conformation and the solvent pH gives rise to an apparent pK_a_ that is within the physiological range. This is a remarkable property because it allows for amino acids that do not normally titrate in this pH range to act as conformational change triggers: if an amino acid has an atypical pKa in one of the conformations then the apparent pKa will be intermediate and may fall in this range.

These conclusions can be extended to other systems of great biochemical interest, where the environment of the amino acid of interest changes significantly with pH. In these systems, where there is a coupling between protonation and conformational states, no possible conclusion can be derived from looking at individual structures and the apparent pK_a_ should be interpreted as the pH at which the conformational change takes place.

## Methods

The initial structures for the CpHMD simulations were built starting from the crystal structures of NO bound NP4 at pH 5.6 (PDB code 1X8O) and at pH 7.4 (PDB code 1X8N) [31]. In these structures, the conformations of the AB and GH loops are characteristic of the closed and open states, respectively. The protonation states of all residues except Asp30 were assigned as suggested from previous experimental [31] and theoretical [33]–[34] studies, and were fixed throughout the simulations. Asp30 was kept as the only titratable residue. This choice is justified by previous studies, which show that CH and Op^−^ are stable conformations while C^−^ and OpH are unstable and begin to undergo the conformational change to the structure that is more stable given the selected protonation state of Asp30 [35] and also, as shown in the results, by the good agreement with the available experimental data.

All simulations were performed at 300 K, maintained using Langevin dynamics with a collision frequency of 2.5 ps^−1^ [65]. The SHAKE algorithm was used to keep bonds involving H atoms at their equilibrium length [66]. Newton's equations were integrated with a time step of 2 fs. The Amber ff99SB force field parameters were used for all residues [67] except the heme, for which parameters developed and tested by our group on previous works were used [68]. The parameters for Asp30 were taken from the original CpHMD article [36]. All simulations were performed with the SANDER module of the AMBER 11 suite [69]. Frames were collected at 1 ps intervals. All simulations were performed using the Generalized Born implicit solvent model [70]. The salt concentration was set at 0.1 M and the cutoff for non-bonded interactions and Born radii was 30 Å.

The CpHMD method [36] was used to allow Asp30 to adapt its protonation state to the specific conformation. This method involves periodic MC sampling of protonation states during the standard generalized Born simulation. At each MC step, a new protonation state is chosen randomly for Asp30 and the free energy of the transition is computed. This value, which depends on both the environment of the titrated residue and the solvent pH, is used as the basis for applying the Metropolis criterion [71] to determine whether the transition will be accepted. If the transition is accepted, the MD simulation continues with Asp30 in the new protonation state. Otherwise, the MD continues with no changes in the protonation state. In this work, a MC step was performed every 20 fs. The pKa value of Asp30 is then computed based on the population of the protonated and deprotonated states using Henderson-Hasselbach's equation.

Four types of CpHMD simulations were performed. Two of these started from the closed conformation, but the solvent pH was set at 5.5 in one case and 7.5 in the other. The other two types were analogous, but started from the open conformation. Also, simulations of the closed structure with a protonated Asp30 and of the open structure with Asp30 ionized were performed for comparative purposes. Over 150 ns of CpHMD simulations were obtained.

## Supporting Information

Figure S1 **AB and GH loops in the context of the complete protein.** A) Closed conformation. B) Open conformation. The AB and GH loops are shown in a different color than the rest of the protein. Leu130 and Asp30 are shown. Asp30 is displayed protonated in the closed structure and deprotonated in the open conformation.
(TIF)

Figure S2 **Val36, Asp35 and Asp129 in the closed and open conformations.** A) Closed conformation. B) Open conformation. Asp 30 is shown in gray for reference. The AB and GH loops are colored according to the conformation. Average values for dCBVal36-CGLeu130 and dCGAsp35-OAsp129 are also shown.
(TIF)

Figure S3 **RMSD of the AB and GH loops.** Running average of the time evolution of the RMSD of the AB and GH loops with respect to the closed (red) and open (black) structures. A) Initially closed structure, simulation at pH 5.5, B) Initially closed structure, simulation at pH 7.5, C) Initially open structure, simulation at pH 5.5, D) Initially open structure, simulation at pH 7.5. In case C the transition to the closed structure takes place almost immediately, but after ∼8 ns the simulation becomes trapped in an intermediate structure (see Text S1 for further detail).
(TIF)

Figure S4 **Time evolution of the Solvent Accessible Surface Area (SASA) of Asp30.** Running average of the time evolution of the SASA of Asp30. A) Initially closed structure, simulation at pH 5.5, B) Initially closed structure, simulation at pH 7.5, C) Initially open structure, simulation at pH 5.5, D) Initially open structure, simulation at pH 7.5. In case C the transition to the closed structure takes place almost immediately, but after ∼8 ns the simulation becomes trapped in an intermediate structure (see Text S1 for further detail).
(TIF)

Figure S5 **Time evolution of relevant parameters when open NP4 is placed at a solvent pH of 5.5.** Top: Running average of distance Asp30-Leu130; Middle: Running average of distance Asp35-Asp129; Bottom: Asp30 microscopic pK_a_. The average values of these distances in the stable simulations of the closed (red) and the open (blue) structures are also shown.
(TIF)

Text S1 **Additional data for transition inducing simulations.** Further details on the simulation of the open conformation at pH 5.5.
(PDF)

Text S2 **Derivations of the equations presented in the main text.** Details of the calculation of the equilibrium concentration of all chemical species and the apparent pK_a_.
(PDF)
