# Supplementary figures and images for: pH-Dependent Conformational Changes in Proteins and Their Effect on Experimental pKas: The Case of Nitrophorin 4
Source: PLoS Comput Biol. 2012 Nov 1;8(11):e1002761. doi: 10.1371/journal.pcbi.1002761 (PMC3486867; doi:10.1371/journal.pcbi.1002761)

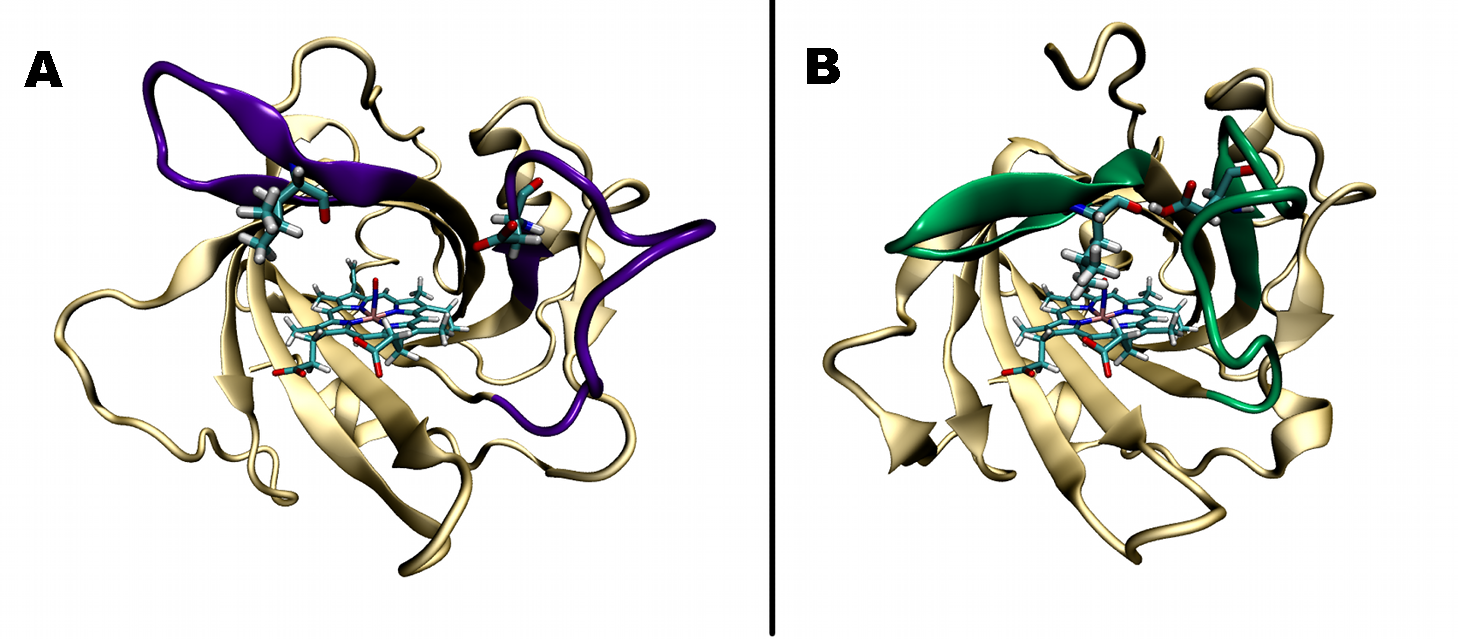

Supplement: Figure S1 — AB and GH loops in the context of the complete protein. A) Closed conformation. B) Open conformation. The AB and GH loops are shown in a different color than the rest of the protein. Leu130 and Asp30 are shown. Asp30 is displayed protonated in the closed structure and deprotonated in the open conformation. (TIF) [file pcbi.1002761.s001.tif]

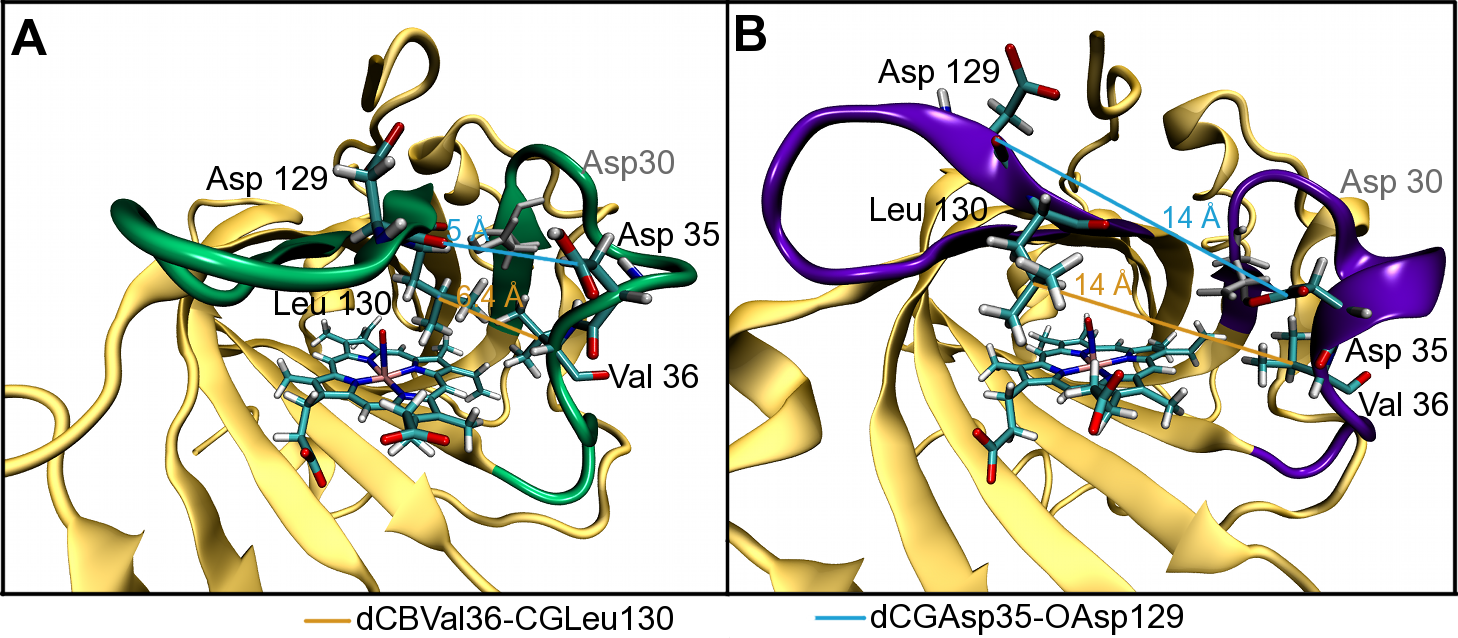

Supplement: Figure S2 — Val36, Asp35 and Asp129 in the closed and open conformations. A) Closed conformation. B) Open conformation. Asp 30 is shown in gray for reference. The AB and GH loops are colored according to the conformation. Average values for dCBVal36-CGLeu130 and dCGAsp35-OAsp129 are also shown. (TIF) [file pcbi.1002761.s002.tif]

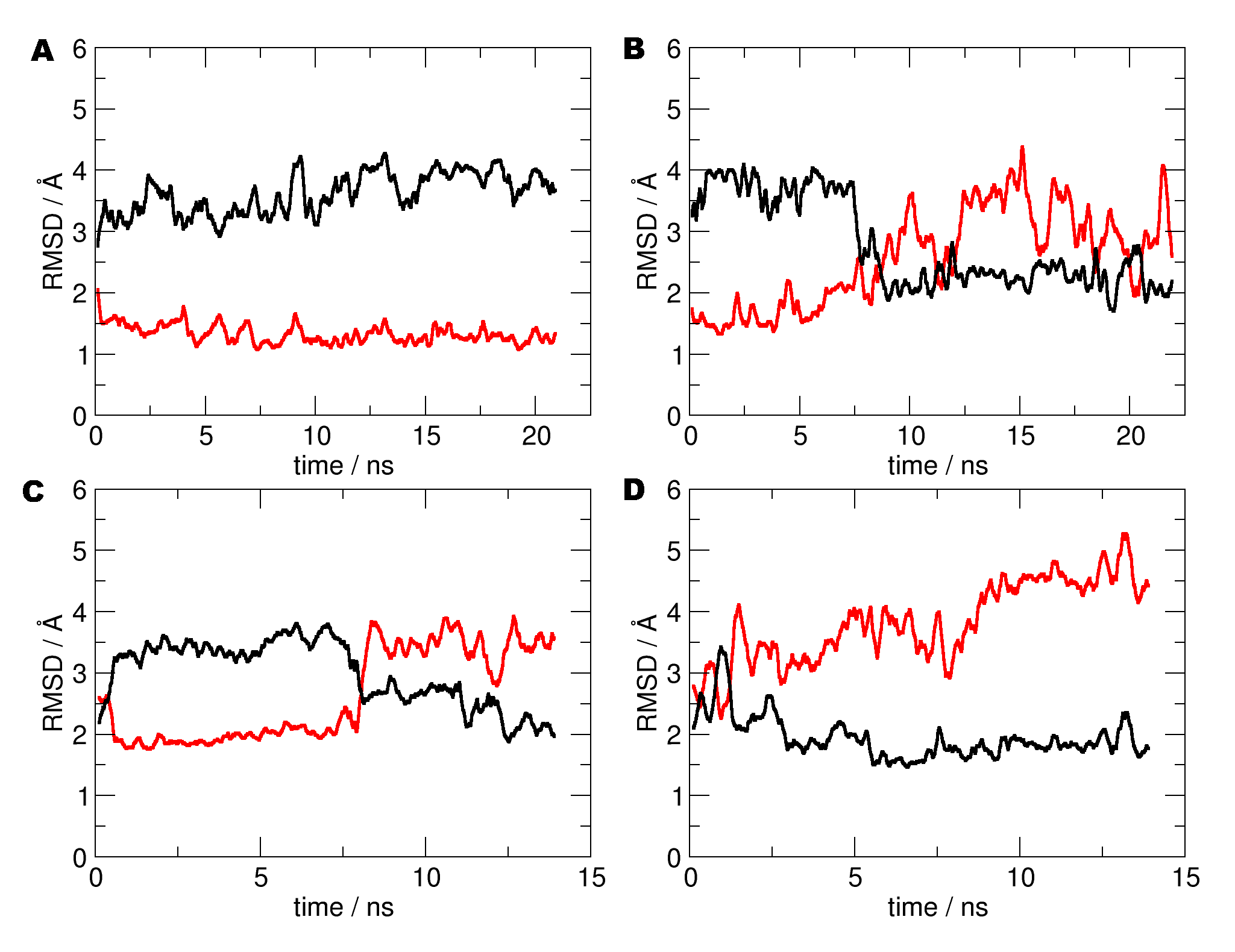

Supplement: Figure S3 — RMSD of the AB and GH loops. Running average of the time evolution of the RMSD of the AB and GH loops with respect to the closed (red) and open (black) structures. A) Initially closed structure, simulation at pH 5.5, B) Initially closed structure, simulation at pH 7.5, C) Initially open structure, simulation at pH 5.5, D) Initially open structure, simulation at pH 7.5. In case C the transition to the closed structure takes place almost immediately, but after ∼8 ns the simulation becomes trapped in an intermediate structure (see Text S1 for further detail). (TIF) [file pcbi.1002761.s003.tif]

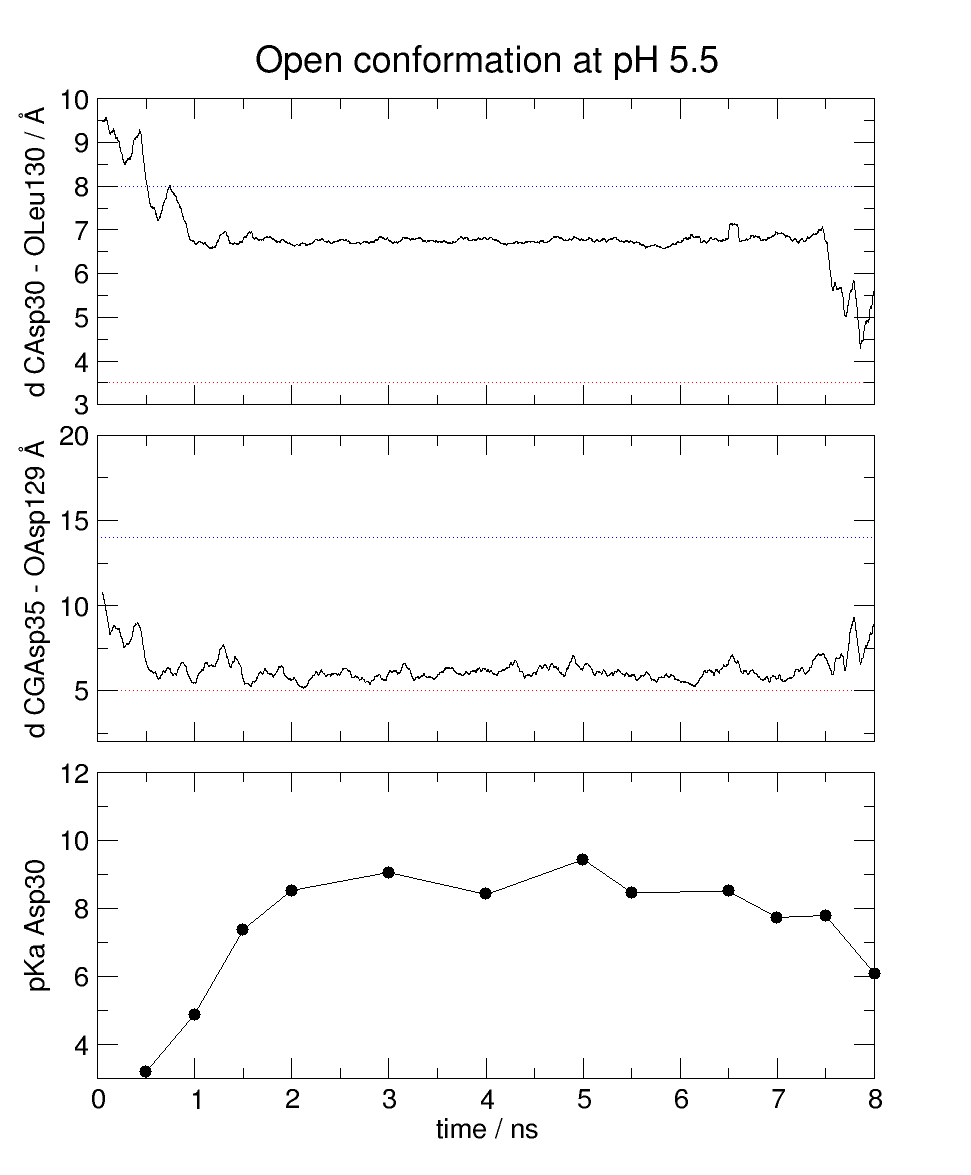

Supplement: Figure S5 — Time evolution of relevant parameters when open NP4 is placed at a solvent pH of 5.5. Top: Running average of distance Asp30-Leu130; Middle: Running average of distance Asp35-Asp129; Bottom: Asp30 microscopic pKa. The average values of these distances in the stable simulations of the closed (red) and the open (blue) structures are also shown. (TIF) [file pcbi.1002761.s005.tif]
