# Supplementary material for: pH-Dependent Conformational Changes in Proteins and Their Effect on Experimental pKas: The Case of Nitrophorin 4
Source: PLoS Comput Biol. 2012 Nov 1;8(11):e1002761. doi: 10.1371/journal.pcbi.1002761 (PMC3486867; doi:10.1371/journal.pcbi.1002761)
Supplement: Text S1 — Additional data for transition inducing simulations. Further details on the simulation of the open conformation at pH 5.5. (PDF) [file pcbi.1002761.s006.pdf]

Supporting Information – Text S1

Additional data for Transition Inducing Simulations: Open conformation at pH 5.5

Rapidly after the simulation starts, Asp 30 becomes protonated and buried inside the protein, with its SASA taking values typical of the closed conformation after 1 ns (Figure S4). This correlates with an increase in the pK_a of Asp30 and a decrease in the distance between CGAsp35 and OAsp129 (Figure S5), which shows that the NO escape pathway is closed. The hydrogen bond between Asp30 and Leu 130 is not formed initially because the carbonyl oxygen of Leu is pointing towards the N-terminus. Instead, Asp 30 is interacting with the carbonyl oxygen of Gly 131 until at 7 ns of simulation a small conformational change positions the Leu oxygen appropriately for the hydrogen bond. Up to this time, however, the interactions present were enough to close the NO escape pathway and bring the AB and GH loops close together as in the closed conformation (Figure S3). The hydrogen bond between Asp30 and Leu130 is not formed long enough to allow the side chains to relax and stabilize the closed conformation. As a result, Asp30 eventually becomes deprotonated and the unfavorable electrostatic interaction pushes the GH loop away. However, the conformation of the loops does not completely match that of the open or the closed conformation (Figure S3), and neither does the SASA (Figure S4).
