# Supplementary material for: pH-Dependent Conformational Changes in Proteins and Their Effect on Experimental pKas: The Case of Nitrophorin 4
Source: PLoS Comput Biol. 2012 Nov 1;8(11):e1002761. doi: 10.1371/journal.pcbi.1002761 (PMC3486867; doi:10.1371/journal.pcbi.1002761)
Supplement: Text S2 — Derivations of the equations presented in the main text. Details of the calculation of the equilibrium concentration of all chemical species and the apparent pKa. (PDF) [file pcbi.1002761.s007.pdf]

Supporting Information – Text S2

Derivation of the equations presented in the paper

Calculation of the equilibrium concentration of all chemical species

The following equilibrium constants are defined based on the thermodynamic cycle shown in Scheme 1:

$$K_{OpH}=\frac{(Op^-)(H^+)}{(OpH)} \quad (1)$$

$$K_{CH}=\frac{(C^-)(H^+)}{(CH)} \quad (2)$$

$$K_H=\frac{(OpH)}{(CH)} \quad (3)$$

$$K_D=\frac{(Op^-)}{(C^-)} \quad (4)$$

Using mole fractions, the following must also be true:

$$(Op^-)+(OpH)+(C^-)+(CH)=1 \quad (5)$$

From the thermodynamic cycle,

$$-RT\ln K_{OpH}=RT\ln K_H-RT\ln K_{CH}-RT\ln K_D \quad (6)$$

Rearranging this equation, it is possible to obtain an expression for K_H as a function of K_D:

$$K_H=\frac{K_{CH}\times K_D}{K_{OpH}} \quad (7)$$

By replacing K_H from equation (7) into equation (3), we get:

$$(OpH)=\frac{(CH)\times K_{CH}\times K_D}{K_{OpH}} \quad (8)$$

Using (8), (4) and (2) into (5), it is possible to obtain an equation for C^- as a function of K_D, (H^+), K_{OpH}, K_{CH}:

$$(C^-)=\frac{1}{1+K_D+(H^+)\left(\frac{K_D}{K_{OpH}}+\frac{1}{K_{CH}}\right)} \quad (9)$$

Then, the mole fraction of all NP4 states can be calculated using equation (9), together with (1), (2) and (4), for a given solvent pH and K_D value:

$$(CH)=\frac{(H^+)}{K_{CH}\left[1+K_D+(H^+)\left(\frac{K_D}{K_{OpH}}+\frac{1}{K_{CH}}\right)\right]} \quad (10)$$

$$(Op^-) = \frac{K_D}{1+K_D+(H^+)\left(\frac{K_D}{K_{OpH}}+\frac{1}{K_{CH}}\right)} \quad (11)$$

$$(OpH)=\frac{K_D}{K_{OpH}\left[1+K_D+(H^+)\left(\frac{K_D}{K_{OpH}}+\frac{1}{K_{CH}}\right)\right]} \quad (12)$$

Calculation of the apparent pK_a

The experimental signal was defined in equation 2 of the paper as

$$S = \alpha([C^-] + [CH]) + \beta([Op^-]+[OpH]) \quad (13)$$

The apparent pK_a is then defined as the inflection point of the S vs. pH curve and can be calculated as the pH for which the second derivative of S equals zero. This calculation was performed using Mathematica (Wolfram Research, Inc., Mathematica, Version 7.0, Champaign, IL (2008)). The obtained apparent pK_a is independent of the constants α and β:

$$\text{apparent } pK_a = pK_{OpH} + pK_{CH} - \log((K_D+1)/(K_D \times K_{CH}+K_{OpH})) \quad (14)$$
